# *Campylobacter* Infections With and Without Bacteremia: A Comparative Retrospective Population-Based Study

**DOI:** 10.1093/ofid/ofae131

**Published:** 2024-03-13

**Authors:** Torgny Sunnerhagen, Rasmus Grenthe, Christian Kampmann, Sara Karlsson Söbirk, Anna Bläckberg

**Affiliations:** Division of Infection Medicine, Department of Clinical Sciences, Lund University, Lund, Sweden; Clinical Microbiology, Infection Prevention and Control, Office for Medical Services, Lund, Sweden; Division of Infection Medicine, Department of Clinical Sciences, Lund University, Lund, Sweden; Department of Infectious Diseases, Skåne University Hospital, Lund, Sweden; Division of Infection Medicine, Department of Clinical Sciences, Lund University, Lund, Sweden; Clinical Microbiology, Infection Prevention and Control, Office for Medical Services, Lund, Sweden; Division of Infection Medicine, Department of Clinical Sciences, Lund University, Lund, Sweden; Department of Infectious Diseases, Skåne University Hospital, Lund, Sweden

**Keywords:** bacteremia, *Campylobacter*, enteritis, outcome

## Abstract

**Background:**

Bacteremia with species in the genus *Campylobacter* is rare, and knowledge of the disease course in comparison with *Campylobacter* enteritis is limited.

**Methods:**

This is a retrospective population-based study. Episodes of *Campylobacter* bacteremia and *Campylobacter* enteritis with a concurrent negative blood culture result that occurred between 2015 and 2022 in southern Sweden were identified through the laboratory database. Medical records were reviewed, and clinical features between patients with bacteremic *Campylobacter* infections were compared with patients with *Campylobacter* spp found in feces.

**Results:**

The study included 29 bacteremic infections with *Campylobacter* and 119 cases of *Campylobacter* spp found in feces. Patients with *Campylobacter* bacteremia were significantly older than those with enteritis (72 years [IQR, 58–62] vs 58 years [IQR, 33–67], *P* < .0001). Eleven patients with bacteremia developed sepsis within 48 hours from blood culturing, and no patient died within 30 days from hospital admission.

**Conclusions:**

*Campylobacter* bacteremia is rare and occurs mainly in the elderly with comorbidities. In comparison with *Campylobacter* infections limited to the gastrointestinal tract, patients with bacteremic *Campylobacter* infections are older and seem more prone to develop sepsis. Classical gastroenteritis symptoms in bacteremic cases with *Campylobacter* may be absent.

*Campylobacter* is a motile gram-negative bacterium with a spiral or curved shape and is known to colonize the gastrointestinal tract of animals, such as wild animals as well as farm and companion animals. Infections due to *Campylobacter* are one of the most common causes of bacterial enteritis globally, and according to the US Centers for Disease Control and Prevention, there are approximately 1.3 million cases of *Campylobacter* infections each year. The main route of transmission causing human *Campylobacter* infections is through consumption of contaminated beef, pork, or poultry [1]. The disease often comprises abdominal pain, fever, and watery and sometimes bloody stools [2, 3]. Postinfectious complications such as reactive arthritis and Guillain-Barré syndrome may also occur [4]. The pathogen rarely enters the bloodstream causing a bacteremic infection. The incidence of *Campylobacter* bacteremia in Scandinavian countries has been estimated to be 2.2 to 2.9 cases per 1 million person-years and to complicate 0.1% to 1% of enteric *Campylobacter* infections [5, 6].

The mortality rates associated with *Campylobacter* bacteremia have been estimated from 2.5% to 15% [5–9]. Bacteremia with *Campylobacter* has typically been observed in patients who are elderly and immunocompromised [8, 10]. However, in a nationwide study from Finland, most patients with *Campylobacter* bacteremia were relatively young and did not have any comorbidities [6]. The species involved in bacteremic *Campylobacter* infections have often been attributed to *C jejuni*, *C coli*, and *C fetus* [6, 9]. Bacteremic episodes with *C fetus* have been associated with a higher age and with a higher rate of endovascular infection as compared with other species within the *Campylobacter* genus [9].

Another Swedish study showed that an increased incidence of *Campylobacter* bacteremia was associated with the change in blood culture bottles, from 32 cases in 2013 to 83 in 2014. These changes coincided with the introduction of BacT/Alert Plus bottles in 2014 [11].

A recent multicentric study from France observed that *Campylobacter* bacteremia most often occurs in immunocompromised cases with a 30-day mortality rate of 11.7%. Also, *C ureolyticus* seemed to be associated with causing deep abscesses [12]. However, in this study, the authors did not compare their sample with patients in whom *Campylobacter* was found only in feces. A multicentric study from Japan investigated 39 patients with *Campylobacter* bacteremia, and just 50% of them presented with either fever or gastrointestinal symptoms [13]. *Campylobacter* bacteremia may not always manifest with typical gastrointestinal symptoms or fever, making diagnosis and differentiation from enteritis more challenging.

This study aimed to investigate the bacteriologic and clinical characteristics of patients with *Campylobacter* bacteremia in comparison with patients with acute enteritis due to *Campylobacter* with a concurrent negative blood culture result.

## METHODS

### Study Cohort and Clinical Setting

All episodes of *Campylobacter* bacteremia that occurred between 2015 and 2022 in southern Sweden were identified through the clinical microbiology laboratory of the Region Skåne and included in the study. This laboratory covers all public and private hospitals in the Region Skåne (approximate population in 2022, 1.4 million) as well as outpatient clinics. All episodes of *Campylobacter* spp in fecal cultures or polymerase chain reaction (PCR) with a concurrent negative blood culture result that occurred between 2015 and 2022 were identified and systemically randomized after being included in the study. The total number of controls per case was 4, and this selection was consistent over the years. Controls for the study were generated from the retrospective cohort of patients with a finding of *Campylobacter* in feces and a concurrent negative blood culture result. To ensure that the selected controls were spread evenly over time, patients were listed according to sampling date, and evenly spaced patients were chosen as control (ie, a selected control for every 11 patients in the potential control group). This was repeated twice so that the control group was approximately 4 times the bacteremia group.

During the study period, the BACTEC FX (Becton Dickinson) blood culture system was used at the clinical microbiology laboratory in the Region Skåne. In fecal samples, *Campylobacter* was detected with selective *Campylobacter* agar (Neogen), with CampyGen (Thermo Scientific) to achieve a microaerophilic environment, from 2017 through February 2020; after that, a PCR-directed method was used to detect DNA by the Amplidiag bacterial GE panel (Mobidiag), targeting the *rimM* and *gyrB* genes of *C jejuni/coli*. After introduction of the molecular method, *Campylobacter* was cultured only from fecal samples on specific requests.

### Data Collection

Medical records of included patients with *Campylobacter* spp in blood cultures or the detection of *Campylobacter* spp in feces (as identified through culture or PCR) were retrospectively reviewed. Demographic and clinical variables were collected and extracted. Comorbidities were assessed according to the Charlson Comorbidity Index [14] and modified according to Quan et al [15]. Laboratory results were collected within 48 hours from blood culture sampling. Any presence of sepsis with or without septic shock within 48 hours from blood culture sampling was assessed according to Sepsis-3 criteria [16] and evaluated according to sequential organ dysfunction score [17].

The presence of *Campylobacter* in samples taken from sterile locations, polymicrobial growth, or serologic testing was noted. Any signs of reactive arthritis after *Campylobacter* infection or gastroenteritis-associated symptoms noted in the medical records, up to 6 months from the first blood culture sampling, were documented. Immunosuppression was defined as at least 5 mg of prednisolone or equivalent cortisol treatment or the use of nonsteroidal immunosuppressant drugs or certain types of biological therapy.

### Statistics

Values are given as median for continuous variables and as proportions for categorical variables. Differences in continuous variables were analyzed by Mann-Whitney *U* test, and Fisher exact test was applied for the analysis of categorical variables. For calculations of incidence rates, the numbers of residents were retrieved from Statistics Sweden via the date of 1 January for the years 2015 to 2022. Analyses were performed with Prism version 9 (GraphPad Software). *P* < .05 was considered statistically significant.

## RESULTS

### Study Cohort

**Figure 1.**
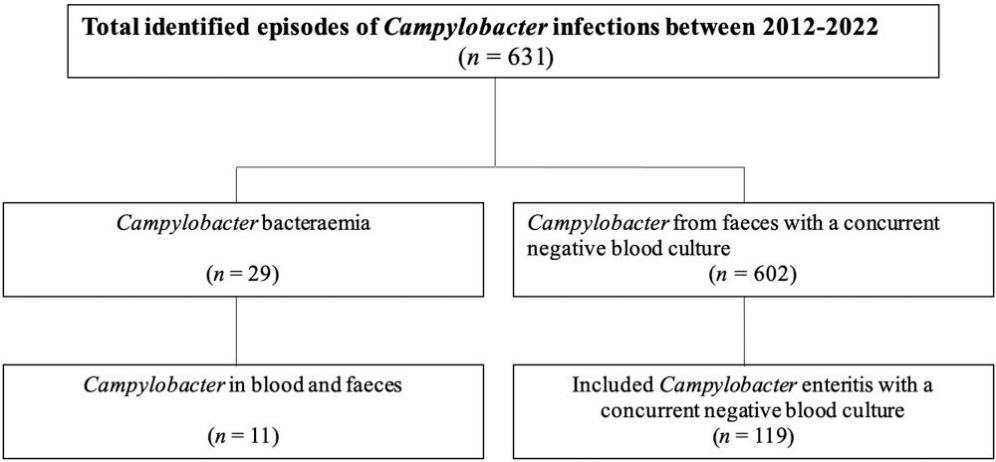
Flow scheme for identification of episodes of campylobacter infections. Controls for the study were generated from the retrospective cohort of patients with a finding of *Campylobacter* in feces and a concurrent negative blood culture result. To ensure that the selected controls were spread evenly over time, patients were listed according to sampling date (ie, 1 selected control for every 11 patients in the potential control group). This was repeated twice so that the control group was approximately 4 times the bacteremia group. As such, the control group used in the study (approximately 20% of all patients with a negative blood culture result) was selected and distributed evenly over time to match the cases with bacteremia.

In total, 631 *Campylobacter* infections, where blood cultures were obtained, occurred in south Sweden during 2015 to 2022. Twenty-nine of these episodes had *Campylobacter* bacteremia and were included in the study. An overall 602 patients with *Campylobacter* in feces with a concurrent negative blood culture result were identified. After systemic randomization and inclusion, 119 of 602 patients were included in the study. Figure 1 summarizes the identification and inclusion of patients with *Campylobacter* infections.

### *Campylobacter* Bacteremia

*C jejuni* and *C coli* were the most frequent species causing *Campylobacter* bacteremia (n = 15), followed by *C ureolyticus* (n = 7), *C fetus* (n = 2), *C curvus* (n = 1), and *C lari* (n = 1). In 3 cases of *Campylobacter* bacteremia, it was not possible for the laboratory to determine the *Campylobacter* species.

The estimated incidence of bacteremic infections with *Campylobacter* was 2.7 per 1 million inhabitants per year. The distribution of *Campylobacter* bacteremia was even throughout the years from 2015 to 2022 (Supplementary Table 1).

### Clinical Features of *Campylobacter* Infections With or Without Bacteremia

Table 1 summarizes the clinical characteristics of the study cohort. The median age was significantly higher in patients with bacteremia (72 years; IQR, 56–82) than in patients with only *Campylobacter* enteritis (51 years; IQR, 33–67; *P* < .0001). Patients with *Campylobacter* bacteremia had significantly more comorbidities (Charlson median score, 2 [IQR, 0.5–5] vs 0 [IQR, 0–1]; *P* < .0001), including malignancy (34% vs 8%, *P* = .0005), renal disease (31% vs 3%, *P* < .0001), and congestive heart failure (21% vs 3%, *P* = .002). Diarrhea was noted for 69% of patients with *Campylobacter* bacteremia, which was significantly lower when compared with patients with *Campylobacter* limited to the gastrointestinal tract (98%, *P* < .0001).

**Table 1.** Clinical Features of *Campylobacter* Bacteremia and Enteritis

	*Campylobacter*, Median (IQR) or No. (%)	
	Bacteremia (n = 29)	Enteritis (n = 119)
Clinical characteristic		
Age, y	72 (56–82)****	51 (33–67)
Gender: male	22 (76)*	60 (50)
Charlson Comorbidity Index	2 (0.5–5)****	0 (0–1)
Congestive heart failure	6 (21)**	3 (3)
Dementia	3 (10)	2 (2)
Chronic pulmonary disease	5 (17)	11 (9)
Rheumatologic disease	3 (10)	8 (7)
Liver disease	1 (3)	3 (3)
Diabetes with chronic complications	3 (10)	5 (4)
Malignancy	10 (34)***	9 (8)
Renal disease	9 (31)****	4 (3)
Immunosuppression	5 (17)	16 (13)
Hemiplegia/paraplegia	1 (3)	0 (0)
Travel abroad	1 (3)***	38 (32)
Known or suspected source of infection	2 (7)***	52 (44)
Animal contact/agriculture stay	2 (7)	8 (7)
Clinical presentation		
Symptom		
Fever	24 (83)	95 (80)
Shaking chills	12 (41)	49 (41)
Abdominal pain	9 (31)****	88 (74)
Vomiting	8 (28)	49 (41)
Diarrhea	20 (69)****	117 (98)
Bloody stool	3 (10)	25 (21)
Inpatient hospital care	28 (97)**	84 (71)
Length of stay, d	7 (5–12)****	4 (3–6)
Antibiotic treatment	28 (97)****	60 (50)
Length, d	13 (9–17)****	0.5 (0–4)
Intravenous	6 (3–8)****	0 (0–2)
Oral	7 (3–10)****	0 (0–0)
Outcome		
Intensive care unit treatment	5 (17)**	1 (1)
Sepsis	6 (21)**	5 (4)
Septic shock	2 (7)*	0 (0)
Mortality rate		
30 d	0 (0)	0 (0)
90 d	4 (14)**	1 (1)
180 d	5 (17)**	3 (3)
1 y	8 (28)***	5 (4)
Laboratory findings		
C-reactive protein, mg/L	173 (111–227)	145 (93–209)
White blood cell count, ×10^9^/L	12 (9–19)***	9 (7–11)

*P < .05. **P < .01. ***P < .001. ****P < .0001.

The median hospital stay was longer for patients with *Campylobacter* bacteremia than for patients with *Campylobacter* enteritis (7 days [IQR, 5–12] vs 4 days [IQR, 3–6], *P* < .0001). Five patients with *Campylobacter* bacteremia were admitted to the intensive care unit, of which 2 patients developed septic shock within 48 hours from sampling for blood culture. There was no case of death within 30 days of the sampling of the first positive test result in the study cohort. Four patients with *Campylobacter* bacteremia died within 90 days from blood culture sampling, as compared with 1 patient with only *Campylobacter* enteritis.

Of the 7 patients with *C ureolyticus*, 4 developed sepsis within 48 hours of blood culture sampling, and 3 of them required treatment at the intensive care unit. Table 2 summarizes the differences between patients with *C jejuni/coli* and those with *C ureolyticus* bacteremia. Patients with *C ureolyticus* required longer hospital stays as well as antibiotic treatment when compared with patients with *C jejuni/coli* (*P* < .01).

**Table 2.** Clinical Differences of *Campylobacter* Bacteremia Depending on Species

	*Campylobacter*, Median (IQR) or No. (%)	
	*C jejuni/coli* (n = 15)	*C ureolyticus* (n = 7)
Clinical characteristic		
Age, y	70 (52–78)	73 (50–82)
Gender: male	4 (27)	5 (71)
Charlson Comorbidity Index	1 (0–3)	4 (2–5)
Congestive heart failure	1 (7)	2 (29)
Dementia	1 (7)	1 (14)
Chronic pulmonary disease	1 (7)	1 (14)
Rheumatologic disease	1 (7)	2 (29)
Liver disease	0 (0)	0 (0)
Diabetes with chronic complications	1 (7)	0 (0)
Malignancy	4 (27)	4 (57)
Renal disease	4 (27)	3 (43)
Immunosuppression	0 (0)	0 (0)
Hemiplegia/paraplegia	0 (0)	0 (0)
Travel abroad	1 (7)	0 (0)
Known or suspected source of infection	2 (13)	0 (0)
Animal contact/agriculture stay	2 (13)	0 (0)
Clinical presentation		
Symptoms		
Fever	14 (93)	5 (71)
Shaking chills	6 (40)	4 (57)
Abdominal pain	6 (40)	3 (43)
Vomiting	4 (27)	2 (29)
Diarrhea	12 (80)	4 (57)
Bloody stool	2 (13)	0 (0)
Inpatient hospital care	14 (93)	7 (100)
Length of stay, d	5 (4–8)**	14 (8–73)
Antibiotic treatment, d	10 (5–13)**	14 (11–68)
Intravenous	3 (2–6)**	7 (4–68)
Oral	6 (3–10)	8 (0–10)
Outcome		
Intensive care unit treatment	2 (13)	3 (43)
Sepsis	0 (0)**	4 (57)
Septic shock	0 (0)	2 (29)
Mortality rate		
30 d	0 (0)	0 (0)
90 d	1 (7)	2 (29)
180 d	1 (7)	2 (29)
1 y	2 (13)	4 (57)
Laboratory findings		
C-reactive protein, mg/L	143 (70–214)	210 (94–301)
White blood cell count, ×10^9^/L	12 (9–18)	12 (11–24)

**P < .01.

Of the 29 patients with *Campylobacter* bacteremia, 18 had fecal samples tested for *Campylobacter* (11 with fecal cultures and 7 with PCR). Eleven patients (61% of those tested, 38% of the total) were positive for *Campylobacter* in feces: 6 from cultures and 5 from PCR. All were positive for *C jejuni/coli*. Patients with *Campylobacter* bacteremia who tested positive for *Campylobacter* in feces had their fecal tests taken at a median 1 day after their blood cultures, as compared with a median 4 days after blood culturing for patients who tested negative (*P* = .05).

### Antibiotic Susceptibility Results

Antibiotic susceptibility results regarding the blood isolates of *Campylobacter* were retrieved. A total of 18 isolates of *C coli* and *C jejuni* had been tested with disk diffusion for erythromycin, ciprofloxacin, and tetracycline according to EUCAST guidelines. Six of these were resistant to ciprofloxacin and 1 toward erythromycin. Nine isolates had been tested under anaerobic conditions for benzylpenicillin, clindamycin, piperacillin-tazobactam, and imipenem via gradient tests. EUCAST has no breakpoint for anaerobic *Campylobacter* species. The minimum inhibitory concentration (MIC) values for these isolates were instead compared with the EUCAST guidance document for species where there are no break points [18]. In this document, MIC value cutoffs are given, above which EUCAST recommends against using the antibiotic in question against anaerobic bacteria without species-specific antibiotic break points. In general, the isolates had MIC values for these antibiotics below these cutoffs. The exception was 1 isolate that had a metronidazole MIC of 8 mg/L, which is above the recommended cutoff of 4 mg/L.

### Antibiotic Treatment

Patients with *Campylobacter* bacteremia most often received initial intravenous empirical treatment with a beta-lactam (n = 27). Sixteen patients received a third-generation cephalosporin, 6 were treated with piperacillin-tazobactam, and 2 each received a carbapenem and penicillin G. Regarding the definite oral antibiotic regimen for patients with *Campylobacter* bacteremia, 5 received ciprofloxacin, 5 were prescribed azithromycin, and 4 were treated with erythromycin. Seven patients were given imidazole-based derivates. Five of these had a bacteremia with anaerobic *Campylobacter*, but 2 had findings of *C jejuni* and *C coli* which are insensitive to imidazole derivatives.

An overall 31 patients with enteritis received antibiotic therapy directed against *Campylobacter* infection. The 3 most frequently administered antibiotics against *Campylobacter* enteritis were fluoroquinolones (n = 13), followed by macrolides (n = 13). Four patients received imidazole-based derivates, which are unlikely to have an effect against *C jejuni* or *C coli*.

## DISCUSSION

This population-based study showed that bacteremic infection due to *Campylobacter* is rare and often occurs in the elderly with comorbidities. As compared with patients with enteritis only, patients with *Campylobacter* bacteremia more often developed sepsis that required intensive care unit treatment. The incidence of bacteremic infections with *Campylobacter* in our study (2.7 per 1 million inhabitants per year) was comparable to that of our neighboring country Denmark, where an incidence of 2.9 per 1 million person-years has been estimated [5]. However, during the study period, the impact of the COVID-19 pandemic may have influenced the results of the total numbers of *Campylobacter* enteritis in the region since traveling during these years did decrease, but bacteremic infection with the pathogen did not seem to be affected. In our study, just 1 patient exhibited symptoms in connection to traveling abroad, which indicates that the bacteremic episodes were often of domestic origin. The mortality rate was low, which is in contrast to a Spanish study by Font et al, which detected a mortality rate of 30% [19]. In contrast to the Finnish study [6] but in line with the Danish study [5], the patients with bacteremic *Campylobacter* infections were older and had more comorbidities, including immunosuppression.

In the patients with bacteremic infection with *Campylobacter*, only 19 were tested for *Campylobacter* in feces, of which 11 samples were positive. It would be interesting to investigate whether there were any differences between bacteremic *Campylobacter* infection with enteritis as compared with bacteremic *Campylobacter* infection with a negative stool analysis. Some potential sources of error explaining why the patients had a negative stool analysis may be inaccurate sampling or inaccurate recordkeeping. These results may also be due to blood culture being more sensitive than fecal culture or to a low and transient bacterial load in the gastrointestinal tract giving rise to bacteremia that was subsequently detected. Just 69% of the patients with bacteremia in our study had diarrheal symptoms. This is in line with some prior studies [8, 9].

Interestingly, we found differences between patients with *C jejuni/coli* bacteremia and those with *C ureolyticus*, where the latter etiology required longer treatment and patients were more prone to develop sepsis and septic shock. While infections with *C ureolyticus* have been described before and are able to translocate through human gastrointestinal epithelium, this is an interesting observation [20–22]. In a large retrospective multicenter study encompassing 592 patients, *C jejuni/coli* and *C fetus* were the most common species, followed by *C ureolyticus*. Confirming our results, immunosuppression and high age were associated with *Campylobacter* bacteremia [12].

In most cases involving *Campylobacter* bacteremia, empirical antibiotic therapy often consisted of a beta-lactam (90%). However, this choice is not optimal as beta-lactams do not target *Campylobacter*. This occurrence may be attributed to the patients presenting with diffuse symptoms, a lack of diarrhea, and an absence of stomach pain, which deviates from the typical presentation of a *Campylobacter* infection. Selecting appropriate empirical therapy for *Campylobacter* infections poses a challenge due to these atypical presentations. Improving diagnostic strategies and considering alternative antibiotic classes, such as fluoroquinolones or macrolides, which may be effective against *Campylobacter*, are crucial steps in optimizing treatment outcomes for patients with *Campylobacter* bacteremia.

The study is limited due to its retrospective study design and the small population size, possibly because we were not able to identify risk factors for developing *Campylobacter* bacteremia when compared with *Campylobacter* enteritis. All patients identified and included in this study had their blood cultures taken at the emergency department. This may have been a sample bias since many patients with *Campylobacter* infection experience mild symptoms and do not require hospital-based care, which may have resulted in our cohort being more fragile, immunocompromised, and older. A major strength of the study is the fact that it is population based and covers all health care centers and hospitals in a defined geographic area.

## CONCLUSION

Bacteremic *Campylobacter* infection is rare and may strike elderly patients with comorbidities. The outcome is favorable, and classical gastroenteritis symptoms may be absent.

## Supplementary Material

ofae131_Supplementary_Data

## Supplementary Data

Supplementary materials are available at *Open Forum Infectious Diseases* online. Consisting of data provided by the authors to benefit the reader, the posted materials are not copyedited and are the sole responsibility of the authors, so questions or comments should be addressed to the corresponding author.

## Notes

***Acknowledgments***. We thank Lena Hyllebusk for her valuable help with the data acquisition

***Author contributions***. R. G. and T. S. extracted data from medical records and performed data acquisition. A. B. and T. S. drafted the manuscript, and A. B. performed data analysis and visualization with C. K. T. S. and A. B. designed the study, with input from S. K. S. T. S., A. B., C. K., R. G., and S. K. S. critically revised the manuscript and approved the final version.

***Data availability***. All relevant data are within the article.

***Patient consent statement***. The study design was approved by the Swedish Ethical Review Authority (2021-04866). Due to the study's observational nature, the need for patient consent was waived.

***Financial support***. This work was supported by the Royal Physiographic Society of Lund to A. B.
