# Supplementary material for: Campylobacter Infections With and Without Bacteremia: A Comparative Retrospective Population-Based Study
Source: Open Forum Infect Dis. 2024 Mar 13;11(3):ofae131. doi: 10.1093/ofid/ofae131 (PMC10960602; doi:10.1093/ofid/ofae131)
Supplement: ofae131_Supplementary_Data [file ofae131_supplementary_data.docx]

**Supplementary Table 1**

| **Year** | ***Campylobacter* bacteraemia (*n*)** |
| --- | --- |
| 2015 | 4 |
| 2016 | 5 |
| 2017 | 2 |
| 2018 | 5 |
| 2019 | 2 |
| 2020 | 4 |
| 2021 | 3 |
| 2022 | 4 |
